# Prevalence and Factors Associated with Parents’ Non-Intention to Vaccinate Their Children and Adolescents against COVID-19 in Latin America and the Caribbean

**DOI:** 10.3390/vaccines9111303

**Published:** 2021-11-09

**Authors:** Diego Urrunaga-Pastor, Percy Herrera-Añazco, Angela Uyen-Cateriano, Carlos J. Toro-Huamanchumo, Alfonso J. Rodriguez-Morales, Adrian V. Hernandez, Vicente A. Benites-Zapata, Guido Bendezu-Quispe

**Affiliations:** 1 Facultad de Ciencias de la Salud, Universidad Científica del Sur, Carrera de Medicina Humana, Lima 15067, Peru; 2 Instituto de Evaluación de Tecnologías en Salud e Investigación—IETSI, EsSalud, Lima 14072, Peru; silamud@gmail.com; 3 Red Internacional en Salud Colectiva y Salud Intercultural, Mexico City 56690, Mexico; guidobq@gmail.com or; 4 Universidad Privada San Juan Bautista, Hospital Nacional Dos de Mayo, Lima 15067, Peru; 5 Medecins Sans Frontieres, Health Politics, 1050 Brussels, Belgium; dra.uyen@gmail.com; 6 Unidad para la Generación y Síntesis de Evidencias en Salud, Universidad San Ignacio de Loyola (USIL), Lima 15012, Peru; ctoro@usil.edu.pe; 7 Clínica Avendaño, Unidad de Investigación Multidisciplinaria, Lima 15074, Peru; 8 Latin American Network of Coronavirus Disease 2019 Research (LANCOVID), Pereira 660003, Colombia; ajrodriguezmmd@gmail.com or; 9 Grupo de Investigación Biomedicina, Faculty of Medicine, Fundación Universitaria Autónoma de las Américas, Pereira 660003, Colombia; 10 Health Outcomes, Policy, and Evidence Synthesis (HOPES) Group, School of Pharmacy, University of Connecticut, Storrs, CT 06269, USA; adrian.hernandez-diaz@uconn.edu; 11 Unidad de Revisiones Sistemáticas y Metaanálisis, Guías de Práctica Clínica y Evaluaciones Tecnológicas Sanitarias, Vicerrectorado de Investigación, Universidad San Ignacio de Loyola (USIL), Lima 15024, Peru; 12 Centro de Investigación Epidemiológica en Salud Global, Universidad Privada Norbert Wiener, Lima 14072, Peru

**Keywords:** COVID-19, SARS-CoV-2, COVID-19 vaccines, vaccination refusal, vaccination, child, adolescent, parents, Latin America

## Abstract

We aimed to estimate the prevalence and factors associated with parents’ non-intention to vaccinate their children and adolescents against COVID-19 in Latin America and the Caribbean (LAC). We performed a secondary analysis using a database generated by the University of Maryland and Facebook (Facebook, Inc., Menlo Park, CA, USA). We included adult (18 and over) Facebook users residing in LAC who responded to the survey between 20 May 2021 and 14 July 2021. We included sociodemographic characteristics, comorbidities, mental health, economic and food insecurity, compliance with mitigation strategies against COVID-19, and practices related to vaccination against this disease. We estimated the crude (cPR) and adjusted (aPR) prevalence ratios with their respective 95%CI. We analyzed a sample of 227,740 adults from 20 LAC countries. The prevalence of parents’ non-intention to vaccinate their children and adolescents against COVID-19 was 7.8% (*n* = 15,196). An age above 35 years old, educational level above college, compliance with physical distancing, use of masks, having economic insecurity, having had COVID-19, anxiety symptoms, depressive symptoms, having a chronic condition or two or more comorbidities, and being vaccinated were associated with a lower prevalence of non-intention to vaccinate children and adolescents against COVID-19. Living in a town, a village, or a rural area was associated with a higher prevalence of non-intention to vaccinate children and adolescents against COVID-19. Approximately nine out of ten parents in LAC intended to vaccinate their children and adolescents against COVID-19. Our results allow for understanding parents’ intentions to vaccinate children and adolescents and help promote and develop education strategies for national vaccination plans against COVID-19.

## 1. Introduction

As of 1 November 2021, more than 246 million confirmed cases of COVID-19 had been reported worldwide with more than 5 million deaths [1]. As for many other infectious diseases, immunization is the most effective public health strategy against the SARS-COV-2 pandemic. It is necessary to reach at least 70% of the population to control this disease [2]. To date, more than 7 billion doses have been administered globally [1].

Given the reticence of vaccination among adults [3,4,5] and other access barriers, it will be necessary to expand vaccination coverage by adding other populations such as children and adolescents, who may constitute a reservoir of the virus and maintain transmission [6]. Although COVID-19 affects children to a lesser extent and severity than adults [2,7], it is known that when community transmission increases, additional deaths occur in children and adolescents [8]. In this regard, clinical trials are currently testing the safety and efficacy of COVID-19 vaccines in adolescents [9]. With the endorsement of some ongoing trials for children [10], some regulatory agencies such as the European Medicines Agency (EMA) and the Food and Drug Administration (FDA) have approved emergency use in adolescents between 12 and 15 years of age, as well other national agencies [11,12]. In this sense, some countries have already started vaccination against COVID-19 in adolescents and considered including children in their schedules [2,13].

Among the multiple barriers to achieve access to vaccination, vaccine reluctance is one of the most complex phenomena and is considered by the WHO as a threat to global health [14]. As in the case of vaccination in adults, parents fear adverse events and have doubts regarding the need to vaccinate their children and adolescents against COVID-19. Nevertheless, previous studies report an intention to vaccinate against COVID-19 in children of between 60% and 70% [15,16,17,18,19]. Various factors related to acceptance of the vaccine in children and adolescents have been described, such as the age of the children, having chronic diseases, having complied with the other vaccines of the immunization schedule, recent vaccination against influenza and if the parents showed concern that their child had COVID-19 and confidence in the vaccine and health institutions, among other factors [15,16,17,18,19,20,21,22].

Latin America is one of the regions most affected by the pandemic. Studies show that LAC countries, although implementing strict control measures against COVID-19 and gradually increasing the capacity of their health systems, experienced many cases and deaths, which in some cases were much higher than the official figures reported [23,24]. This was because pre-pandemic conditions in their health systems and social determinants [21,22] undermined the effectiveness of their responses. Likewise, there was no comprehensive strategy for testing, monitoring and tracking the cases, which contributed to the fact that the spread of the virus was not adequately contained [23,24]. Similarly, economic support measures were implemented late for most countries [23,24].

Likewise, there are problems in the availability of vaccines, which means that in July 2021, some countries only had vaccines available for 3% of their populations [25]. Despite being a global phenomenon, the factors associated with vaccine reticence vary according to the context and characteristics of the population group evaluated [26,27]. Therefore, regional studies involving different populations are necessary to generate specific campaigns [20]. Taking this into account, and given the need to better understand the phenomena related to the acceptance of the vaccine in various population groups in our context, the objective of this study was to estimate the prevalence and factors associated with parents’ non-intention to vaccinate their children and adolescents against COVID-19 in Latin America and the Caribbean (LAC).

## 2. Materials and Methods

### 2.1. Study Design

We performed a secondary analysis using a database generated by the University of Maryland and Facebook (Facebook, Inc., Menlo Park, CA, USA). Both institutions designed a survey to assess sociodemographic characteristics, comorbidities, mental health, economic and food insecurity, compliance with mitigation strategies against COVID-19 and practices related to vaccination against this disease. The survey has been carried out daily since 23 April 2020 in more than 200 countries and in the primary language of the territory. The proportion of Facebook users who are ineligible to participate in the survey due to language and geographic restrictions is less than 5% globally (people for whom it is not possible to ensure that the entire communication from invitation to the study survey is in the user’s language). The surveys are daily repeated cross-sections, including participants that are Facebook users with similar characteristics across each day. The sampling frame for the random selection of participants included the total of Facebook users over 18 years of age from a particular region and country daily. Stratified random sampling using the administrative boundaries within countries or territories is conducted to provide geographic coverage. The selection of the surveyed participants was random based on the sampling frame, which was recalculated daily. If a Facebook user refused to participate, another was randomly invited within the sampling frame. The participants could only answer the survey once within an 8-week time frame. This survey has been used to develop previous studies [22], and the survey methodology has been previously described in greater detail [28]. We have attached in Supplementary Materials the questionnaire used.

### 2.2. Population and Sample

**Figure 1:**
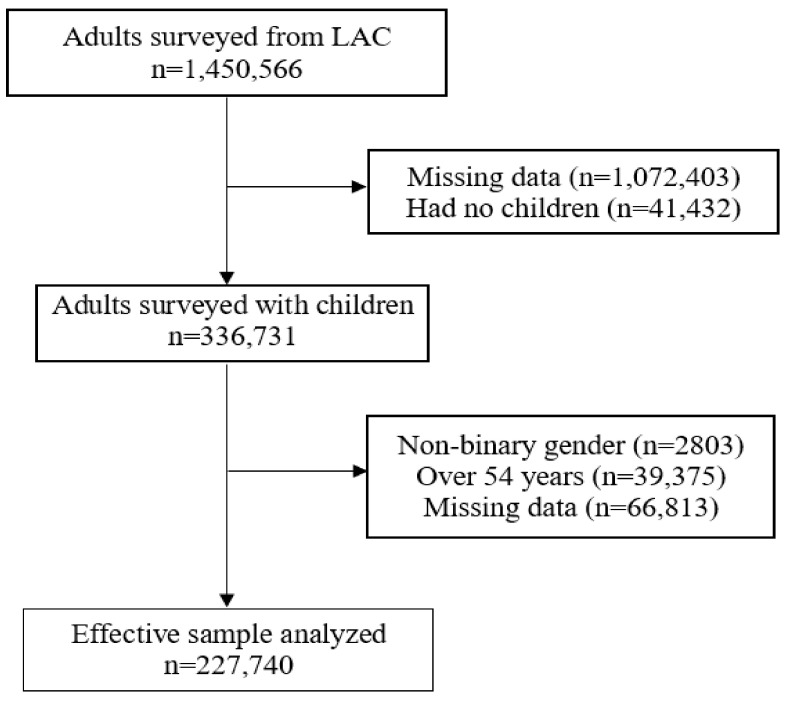
Flowchart of the selection of the study sample.

We included adult (18 and over) Facebook users residing in LAC who responded to the survey between 20 May 2021 and 14 July 2021. We excluded participants who did not have the variables of interest, did not have children, were of non-binary gender and were over 54 years of age. We excluded participants over 54 years of age to reduce the probability of not exclusively including parents of children under 18. Thus, we analyzed 227,740 adults from LAC (Figure 1).

### 2.3. Variables and Measures

#### 2.3.1. Outcome Variable: Parents’ Non-Intention to Vaccinate Their Children and Adolescents against COVID-19

We evaluated the parents’ intention to vaccinate their children and adolescents against COVID-19 using the following survey question: Will you choose to get a COVID-19 vaccine for your child or children when they are eligible? This question had four possible alternatives: yes, definitely; yes, probably; no, probably not; and no, definitely not. Subsequently, we dichotomized the variable considering the first two alternatives for the parents’ intention to vaccinate their children and adolescents, while the last two were considered non-intention.

#### 2.3.2. Independent Variables

Sociodemographic Variables: We included the following sociodemographic variables (in parenthesis is the described the survey question related to the study variables and the categories considered for these variables in the study): gender (What is your gender: male or female?); age (What is your age: 18–24, 25–34, 35–44 or 45–54 years?); educational level (What is the highest level of education that you have completed: university post-graduate degree completed, university completed, college or pre-university; secondary school or high school (or equivalent) completed; primary school completed, less than primary school or no formal schooling?); and area of residence (Which of these best describes the area where you are currently staying: city, town, village or rural area?). We defined a town as a populated area with fixed boundaries and a local self-government, a city as an important or large town and a village as a group of houses and other buildings—usually in the countryside—that is smaller than a town.

Comorbidities, Personal, and COVID-19 History: The participants self-reported the following comorbidities (survey question: Have you ever been told by a doctor, nurse or other health professional that you have any of the following medical conditions?): asthma, chronic obstructive pulmonary disease (COPD) or chronic bronchitis or emphysema, cancer, diabetes, high blood pressure, kidney disease, a compromised or weakened immune system, heart attack or another heart disease and obesity. We generated a variable that grouped the comorbidities as 0, 1, 2, or more. We also included self-reporting of being a smoker (yes or no), having had COVID-19 (yes or no) and having been vaccinated against COVID-19 (yes or no).

Compliance with Community Mitigation Strategies: The community mitigation strategies included were physical distancing and a mask during the last 7 days. We defined physical distancing as a participant reported having intentionally avoided contact with other people at some point in the last 7 days (survey question: In the past 7 days, how often did you intentionally avoid contact with other people?). In addition, the use of masks was defined as whether a participant reported wearing a mask in public at some point during the last 7 days (survey question: In the past 7 days, how often did you wear a mask when in public?).

Food and Economic Insecurity: We assessed food insecurity with the following survey question: How worried are you about having enough to eat in the next week? This question had 4 possible answers: very worried, somewhat worried, not too worried and not worried at all. We considered the first three responses as food insecurity.

We defined economic insecurity using the following survey question: How worried are you about your household’s finances in the next month? It had 4 possible responses: very worried, somewhat worried, not too worried and not worried at all. We defined economic insecurity using the first three responses.

Anxiety and Depressive Symptoms: We evaluated anxiety symptoms using the survey question: During the last 7 days, how often did you feel so nervous that nothing could calm you down? This question is part of the Kessler Psychological Distress Scale (K10), and the survey had 5 possible responses: all the time, most of the time, some of the time, a little of the time and none of the time. Therefore, we dichotomized the variable by considering the first 3 alternatives as the presence of anxiety symptoms.

We evaluated depressive symptoms using the following survey question: How often did you feel so depressed that nothing could cheer you up in the past 7 days? This question is part of the Kessler Psychological Distress Scale (K10), and they survey had 5 possible responses: all the time, most of the time, some of the time, a little of the time and none of the time. Therefore, we dichotomized the variable by considering the first 4 alternatives as the presence of depressive symptoms.

### 2.4. Statistical Analysis

We downloaded the databases in Microsoft Excel 2016^®^ format and imported them into the statistical package Stata/SE ^®^ version 17.0 (StataCorp, College Station, TX, USA). Then, we performed the statistical analysis while considering the complex sampling of the survey and the svy command.

We utilized weighted proportions with their respective 95% confidence intervals (95%CI) and absolute frequencies to present the qualitative variables. We used the Chi-square test with Rao-Scott correction to perform the bivariate analysis between the independent variables and the parents’ non-intention to vaccinate their children and adolescents against COVID-19. We used two generalized linear models (crude and adjusted) of the Poisson family with a logarithmic link function to estimate the factors associated with the parents’ non-intention to vaccinate their children and adolescents against COVID-19. We estimated the crude (cPR) and adjusted (aPR) prevalence ratios with a 95%CI. We elaborated a crude model and an adjusted statistical model (we included the variables with a *p* value < 0.05 in the crude model). We evaluated the possible collinearity of the associated factors included in the final adjusted model.

### 2.5. Ethical Considerations

The participants who responded to the survey provided informed consent. This study analyzed a secondary database that collected data without identifiers and did not violate the integrity of the participants. Access to the database was given with permission from the University of Maryland.

## 3. Results

### 3.1. Characteristics of the Study Sample

**Figure 2:**
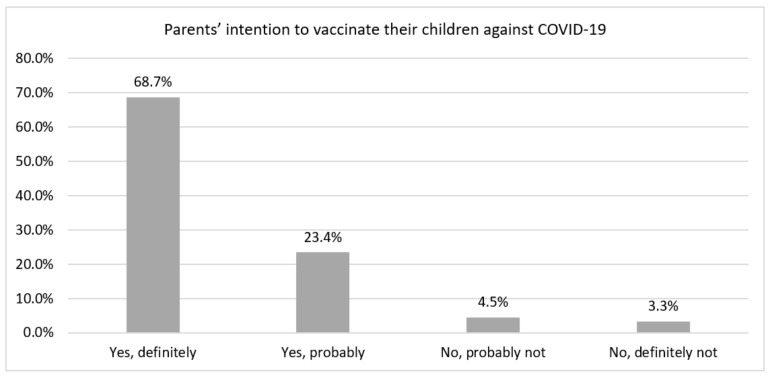
Frequency of parents’ answers about intention to vaccinate their children against COVID-19 in Latin America and the Caribbean countries.

We analyzed a sample of 227,740 adults from 20 LAC countries between 20 May and 14 July 2021. We found that 55.0% (*n* = 140,355) were female, 33.8% (*n* = 86,869) were between 35 and 44 years, 47.3% (*n* = 99,228) had completed primary education or less, and 75.3% (*n* = 182,980) lived in a city. In addition, 90.6% (*n* = 208,183) and 93.4% (*n* = 214,204) had complied with physical distancing and the use of a mask, respectively. We found that 71.1% (*n* = 154,833) reported food insecurity, while 86.7% (*n* = 195,130) reported economic insecurity. In addition, 41.4% (*n* = 98,562) and 45.9% (*n* = 108,557) reported having anxious and depressive symptoms, respectively. Furthermore, 60.9% (*n* = 134,454) of the participants did not report comorbidities, and 66.8% (*n* = 141,615) were not yet vaccinated against COVID-19, while 31.3% (*n* = 70,494) had had COVID-19 at some point, and 7.8% (*n* = 15,196) reported that they had no intention to vaccinate their children and adolescents against COVID-19 (Table 1). In addition, we reported the parents’ answers about intention to vaccinate their children against COVID-19 (Figure 2).

### 3.2. Bivariate Analysis According to Parents’ Non-Intention to Vaccinate Their Children and Adolescents against COVID-19

We found statistically significant differences in the bivariate analysis between the independent variables and the parents’ non-intention to vaccinate their children and adolescents against COVID-19, except for smoking (*p* = 0.481) and food insecurity (*p* = 0.070). The analysis is detailed in Table 2.

### 3.3. Prevalence of Parents’ Non-Intention to Vaccinate Their Children and Adolescents against COVID-19 According to Each LAC Country

**Figure 3:**
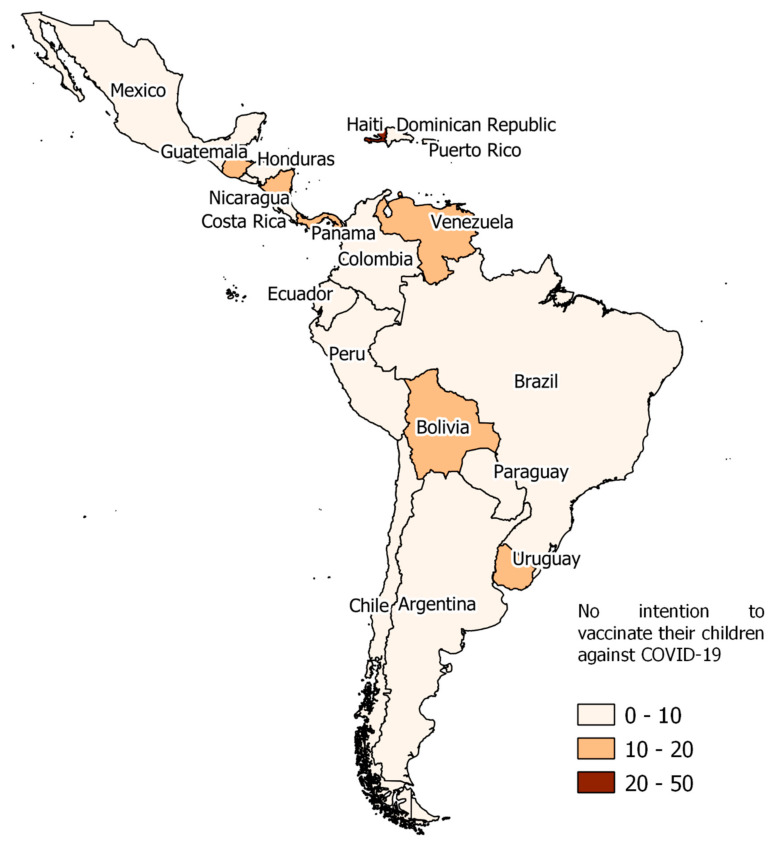
Prevalence of parents’ non-intention to vaccinate their children against COVID-19 in Latin America and the Caribbean countries.

We found that the countries with the highest prevalence of parents’ non-intention to vaccinate their children and adolescents against COVID-19 were Haiti (*n* = 142; 50.0%), Bolivia (*n* = 565; 13.8%), Panama (*n* = 157; 13.7%), Uruguay (*n* = 397; 12.9%) and Nicaragua (*n* = 217; 12.9%). On the other hand, the countries with the lowest prevalence of parents’ non-intention to vaccinate their children and adolescents against COVID-19 were Mexico (*n* = 2200; 5.4%), Honduras (*n* = 113; 5.9%), Brazil (*n* = 4409; 6.1%), El Salvador (*n* = 180; 6.8%), and Peru (*n* = 531; 7.3%) (Figure 3 and Supplementary Materials).

### 3.4. Factors Associated with Parents’ Non-Intention to Vaccinate Their Children and Adolescents against COVID-19

The adjusted statistical regression model showed a lower prevalence of non-intention to vaccinate children and adolescents against COVID-19 among parents between 35 and 44 years old (aPR = 0.86; 95%CI: 0.78–0.94; *p* = 0.001) and 45 to 54 years (aPR = 0.88; 95%CI: 0.80–0.97; *p* = 0.011) compared to those between 18 and 24. We found a higher prevalence of non-intention to vaccinate children and adolescents in parents with college, university, and post-graduate studies (aPR = 1.20; 95%CI: 1.10–1.31; *p* < 0.001), compared to the group without formal schooling, complete or incomplete elementary school. Physical distancing was the practice that was most strongly associated with the outcome (aPR = 0.46; 95%CI: 0.42–0.50; *p* < 0.001). Likewise, use of masks (aPR = 0.66; 95%CI: 0.61–0.70; *p* < 0.001), having economic insecurity (aPR = 0.80; 95%CI: 0.75–0.85; *p* < 0.001), anxiety symptoms (aPR = 0.87; 95%CI: 0.82–0.92; *p* < 0.001), depressive symptoms (aPR = 0.93; 95%CI: 0.87–0.99; *p* = 0.030) and having had COVID-19 (aPR = 0.93; 95%CI: 0.87–0.99; *p* = 0.045) were associated with a lower prevalence of parents’ non-intention to vaccinate their children and adolescents against COVID-19. Additionally, compared to not having comorbidities, having a chronic condition (aPR = 0.82; 95%CI: 0.77–0.87; *p* < 0.001) or two or more comorbidities (aPR = 0.87; 95%CI: 0.81–0.95; *p* = 0.001) was associated with a lower prevalence of parents’ non-intention to vaccinate their children and adolescents against COVID-19. Being vaccinated (aPR = 0.26; 95%CI: 0.23–0.28; *p* < 0.001) was also strongly associated with a lower prevalence of parents’ non-intention to vaccinate their children and adolescents against COVID-19. Conversely, compared to living in a city, living in a town (aPR = 1.29; 95%CI: 1.12–1.48; *p* < 0.001), a village or rural area (aPR = 1.34; 95%CI: 1.22–1.47; *p* < 0.001) was associated with a higher prevalence of parents’ non-intention to vaccinate their children and adolescents against COVID-19 (Table 3).

## 4. Discussion

We found that 9 out of 10 parents in Latin America intended to vaccinate their children or adolescents against COVID-19. This prevalence was higher than that reported in previous studies on this topic. For example, in Canada, a study carried out on parents of children between 9 and 12 years old reported an intention to vaccinate of 60.4% [18]. In Turkey, the intention to vaccinate was as high as 56.8%, with a preference for a locally developed over foreign vaccine [17]. In Switzerland, 59.2% of respondents indicated their intention to vaccinate their children once the vaccine was available [19]. In China, vaccine acceptability for children was 72.6% [16]. A multinational study in the United States, Canada, Israel, Spain, and Switzerland reported that 65% of parents intended to vaccinate their children against COVID-19 [20]. Another multinational study that included Latin American countries, such as Peru, Brazil, Mexico, Argentina, Colombia and Chile, found that 69.2% intended to vaccinate their children [15]. However, these results were not comparable with ours for various reasons, such as the time the study was carried out and the representativeness of the chosen sample. Indeed, the Canadian [18], Chinese and both multinational studies [15,20] were carried out in 2020 before vaccination in children and adolescents was a real possibility. One of the multinational studies only included mothers, whether pregnant or not [15]. The other included only parents who attended emergency departments of hospitals in the countries studied [20]. The Chinese study only included factory workers [16], and the Swiss study only included parents from two regions of the country [19].

Concerning parents between 35 and 54 years old having a greater intention to vaccinate their children, some studies have shown that the older persons are, the more likely they are to accept the COVID-19 vaccine [5]. This finding could be related to the fact that this age group is more likely to have children or adolescents than those over 54 years of age, while those under 35 are more likely to have young children and not consider it necessary or relevant to vaccinate them against COVID -19. Likewise, it was found that parents with comorbidities or health problems such as depression or anxiety had a greater intention to vaccinate their children. In this regard, given the known association between comorbidities and worse clinical outcomes in patients with COVID-19 [29], it is expected that a parent with these clinical conditions may have a higher assessment of the usefulness of vaccines, as they are a group vulnerable to this sickness.

Adherence to maintaining physical distance or wearing a mask and being vaccinated against COVID-19 were associated with a greater intention to vaccinate children against COVID-19. These findings suggest that people adhering to measures based on nationally and globally recommended evidence would follow vaccination recommendations for their children in the same way, indicating family vaccination intention. This correlates with what was found in the multinational study in 16 countries in pregnant women and mothers, which described that the use of face masks was one of the predictors for greater acceptance of the COVID-19 vaccine [15]. In adults, other studies have also shown that the use of masks and social distancing and anxiety were also strongly associated with getting vaccinated [30,31]. Compliance with these community mitigation measures and acceptance of the vaccine for their children may be related to concern about the disease in general and parents’ fear of the consequences of infection for their children [20].

Regarding the parents’ residence, living in a village, town or rural area was associated with a higher probability of non-intention to vaccinate children than parents living in a city. Unfortunately, no study has evaluated the differences regarding the parents’ place of residence. However, a lower intention and vaccination coverage against COVID-19 has been described in adults and pregnant women residing in rural areas [32,33]. Likewise, a previous study described lower vaccination coverage against the human papillomavirus in adolescents in rural areas of the United States [34], suggesting the presence of greater reluctance toward the vaccine against COVID-19 in these areas [35]. In addition, keeping children up to date in the vaccination schedule and having a caregiver vaccinated against influenza has been associated with a greater intention to vaccinate children against COVID-19 [20]. Therefore, vaccination coverage against COVID-19 in adolescents could be improved by increasing vaccination intention in parents residing in rural areas. We found that the parents’ having high educational levels was associated with a greater probability of non-intention to vaccinate their children and adolescents from LAC. This finding is consistent with a previous study carried out in China, which found that parents with college educations or below had a higher acceptance of COVID-19 vaccines, compared with parents with master’s diploma or above [36]. However, this does not agree with a study that evaluated the HPV vaccine hesitancy among parents in Italy, which found no difference between knowing that the HPV vaccine was a preventive measure for this disease and educational level [37]. This could be explained by an infodemic that occurred in LAC countries during 2020 [38,39] and could equally affect people of different educational levels.

It was found that financially insecure parents were more likely to vaccinate their children than those who were not. This result was contrary to various studies showing that parents with low education and income were less likely to intend to vaccinate their children [18]. The higher the income, the greater the probability of accepting vaccination against COVID -19 [40,41]. However, this might be explained by persons with economic insecurity having been more vulnerable to COVID-19 in the region [42]. Those who live with a job and social insecurity are at greater risk of presenting other infections during the pandemic [43]. In this sense, it has been described that people living in areas highly affected by the pandemic have a greater intention to be vaccinated [44], which could support our findings.

Regarding the differences in the non-intention to vaccinate among the countries, differences in the epidemiological situation, the wave or time of the pandemic in which they were found, and the proportion of people affected by COVID-19 in these countries must be taken into account. Likewise, the circulation of variants of the SARS-CoV-2 virus of the most significance, such as the alpha, beta, gamma, and delta variants [45], and especially the latter, could influence the decision to get vaccinated. As of 4 August 2021, this variant is present in 24 countries in the Americas region, and due to its greater transmissibility, mortality from this disease could increase, and health systems could collapse [46].

We must emphasize that the associated factors have different magnitudes, and their interpretation is relevant to prioritize the interventions to be carried out to increase the intention of vaccination in children and adolescents. Then, we highlight the im-portance of some factors that could improve vaccination intention in children, such as compliance with physical distancing and the use of masks, as well as vaccination by parents. On the other hand, they should encourage vaccination in children living in rural areas, as well as in the highest educational levels.

## 5. Limitations

This study has limitations. The respondents were users of a network (Facebook), which implies that the data obtained are from people with internet access and social networks and do not represent a population without these characteristics. Additionally, we did not have the non-response rate, which is relevant within the context of an online survey. Likewise, the variables included in this analysis were those that were available in the survey database, as there may have been other associated factors that were not measured by the survey and should be included in future studies. There is the possibility of hidden bias due to unmeasured confusion (i.e., there may have been some other associated factors that were not measured by the survey and should be included in future studies). Additionally, the data were obtained by self-reporting, and therefore, there may have been an underreporting of information. In addition, due to the study’s design, our results should be interpreted only in the context of associations; causality among the variables evaluated could not be established. Nonetheless, this study presents the strength of analyzing a database with a large representative sample of social network users widely used in LAC countries.

## 6. Conclusions

In conclusion, 9 out of 10 parents in LAC intended to vaccinate their children and adolescents against COVID-19. The intention to vaccinate their children was more remarkable in parents residing in a city and those presenting good adherence to health recommendations or chronic health conditions. In LAC countries, the population of children and adolescents represents a considerable proportion of the population, and despite not being a priority for being among the groups most at risk of severe disease due to COVID-19, they do play an essential role from the point of view of transmission and a source of new infections. It has been estimated that 70% vaccination coverage is necessary to achieve group or herd immunity and to be able to control the pandemic, and this fundamentally depends on vaccination rather than seroprevalence [47]. In this sense, the vaccination of children can play an important role in achieving this coverage [48]. For this reason, favoring vaccination in these groups and understanding the intention of parents to vaccinate is of great importance in developing possible promotion and education strategies for national vaccination plans against COVID-19.

## Figures and Tables

**Table 1:** Descriptive analysis of the study sample (*n* = 227,740; N = 3,378,612).

Characteristics	Total		
	Absolute Frequency of Participants Surveyed	Weighted Proportion According to Each Category	
	*n*	%	95%CI
Gender			
Male	87,385	45.0	44.1–45.8
Female	140,355	55.0	54.2–55.9
Age (years)			
18–24	20,306	11.5	11.0–12.1
25–34	65,107	29.8	29.1–30.5
35–44	86,869	33.8	33.1–34.6
45–54	55,458	24.8	24.2–25.4
Educational level			
Primary school completed, less than primary school or no formal schooling	99,228	47.3	45.9–48.7
Secondary school completed or high school (or equivalent) completed	24,329	12.2	10.4–14.2
University post-graduate degree completed, university completed, college or pre-university	104,183	40.5	38.5–42.5
Living area			
City	182,980	75.3	71.4–78.8
Town	27,981	15.4	12.7–18.5
Village or rural area	16,779	9.3	8.2–10.6
Smoking			
No	187,072	82.7	81.2–84.2
Yes	40,668	17.3	15.8–18.8
Compliance with physical distancing			
No	19,557	9.4	8.9–10.0
Yes	208,183	90.6	90.0–91.1
Compliance with mask use			
No	13,536	6.6	6.2–7.1
Yes	214,204	93.4	92.9–93.8
Food insecurity			
No	72,907	28.9	28.0–29.8
Yes	154,833	71.1	70.2–72.0
Economic insecurity			
No	32,610	13.2	12.7–13.9
Yes	195,130	86.7	86.1–87.3
Anxiety symptomatology			
No	129,178	58.6	57.2–59.9
Yes	98,562	41.4	40.1–42.8
Depressive symptomatology			
No	119,183	54.1	52.8–55.4
Yes	108,557	45.9	44.6–47.2
Comorbidities			
0	134,454	60.9	60.1–61.7
1	61,460	26.1	25.6–26.6
2 or more	31,826	13.0	12.3–13.8
Vaccinated			
No	141,615	66.8	64.5–69.0
Yes	86,125	33.2	31.0–35.5
Had COVID-19			
No	157,246	68.7	67.4–70.0
Yes	70,494	31.3	30.0–32.6
Parents’ intention to vaccinate their children against COVID-19			
Yes	212,544	92.2	91.4–92.9
No	15,196	7.8	7.1–8.6

95%CI: 95% confidence intervals.

**Table 2:** Characteristics according to parents’ intention to vaccinate their children against COVID-19 (*n* = 227,740; N = 3,378,612).

Characteristics	Parents’ Intention to Vaccinate Their Children against COVID-19						
	Yes			No			
	Absolute frequency of participants surveyed	Weighted proportion according to each category		Absolute frequency of participants surveyed	Weighted proportion according to each category		*p* Value
	*n*	%	IC95%	*n*	%	IC95%	
Gender							<0.001
Male	81,164	91.6	90.8–92.3	6221	8.4	7.7–9.2	
Female	131,380	92.6	91.8–93.4	8975	7.4	6.6–8.2	
Age (years)							<0.001
18–24	18,612	89.9	88.6–91.2	1694	10.1	8.8–11.4	
25–34	60,223	91.1	90.1–92.0	4884	8.9	8.0–9.9	
35–44	81,408	92.7	91.9–93.4	5461	7.3	6.6–8.1	
45–54	52,301	93.8	93.2–94.2	3157	6.2	5.8–6.8	
Educational level							0.038
Primary school completed, less than primary school or no formal schooling	92,408	92.2	91.5–92.9	6820	7.8	7.1–8.5	
Secondary school completed or high school (or equivalent) completed	22,348	91.1	90.0–92.1	1981	8.9	7.9–10.0	
University post-graduate degree completed, university completed, college or pre-university	97,788	92.4	91.4–93.3	6395	7.6	6.7–8.6	
Living area							<0.001
City	171,517	92.9	92.3–93.5	11,463	7.1	6.5–7.7	
Town	25,732	90.1	88.4–91.6	2249	9.9	8.4–11.6	
Village or rural area	15,295	89.4	88.1–90.5	1484	10.6	9.5–11.9	
Smoking							0.481
No	174,613	92.1	91.3–92.9	12,459	7.9	7.1–8.7	
Yes	37,931	92.3	91.5–93.1	2737	7.7	6.9–8.5	
Compliance with physical distancing							<0.001
No	16,676	84.0	82.8–85.1	2881	16.0	14.9–17.2	
Yes	195,868	93.0	92.2–93.7	12,315	7.0	6.3–7.8	
Compliance with mask use							<0.001
No	11,976	85.8	84.3–87.2	1560	14.2	12.8–15.7	
Yes	200,568	92.6	91.9–93.3	13,636	7.4	6.7–8.1	
Food insecurity							0.070
No	67,862	91.8	91.0–92.6	5045	8.2	7.4–9.0	
Yes	144,682	92.3	91.5–93.0	10,151	7.7	7.0–8.5	
Economic insecurity							<0.001
No	29,957	90.2	89.3–91.0	2653	9.8	9.0–10.7	
Yes	182,587	92.5	91.7–93.2	12,543	7.5	6.8–8.3	
Anxiety symptomatology							<0.001
No	119,496	91.4	90.6–92.1	9682	8.6	7.9–9.4	
Yes	93,048	93.2	92.4–94.0	5514	6.8	6.0–7.6	
Depressive symptomatology							<0.001
No	110,362	91.5	90.7–92.1	8821	8.5	7.9–9.3	
Yes	102,182	93.0	92.1–93.8	6375	7.0	6.2–7.9	
Comorbidities							<0.001
0	124,340	91.1	90.1–91.9	10,114	8.9	8.1–9.9	
1	58,034	93.8	93.1–94.3	3426	6.2	5.7–6.9	
2 or more	30,170	94.1	93.4–94.7	1656	5.9	5.3–6.6	
Vaccinated							<0.001
No	128,631	89.6	88.5–90.6	12,984	10.4	9.4–11.5	
Yes	83,913	97.4	97.1–97.5	2212	2.6	2.5–2.9	
Had COVID-19							0.015
No	146,727	91.9	91.0–92.8	10,519	8.1	7.2–9.0	
Yes	65,817	92.7	92.1–93.2	4677	7.3	6.8–7.9	

95%CI: 95% confidence intervals.

**Table 3:** Factors associated with parents’ non-intention to vaccinate their children against COVID-19.

Characteristics	Parents’ Non-Intention to Vaccinate Their Children against COVID-19					
	Crude			Statistical Adjusted Model		
	cPR	95%CI	*p* value	aPR	95%CI	*p* Value
Gender						
Male	Reference	-	-	Reference	-	-
Female	0.88	0.82–0.94	<0.001	0.97	0.92–1.03	0.308
Age (years)						
18–24	Reference	-	-	Reference	-	-
25–34	0.89	0.82–0.96	0.002	0.94	0.88–1.02	0.137
35–44	0.73	0.66–0.80	<0.001	0.86	0.78–0.94	0.001
45–54	0.62	0.56–0.69	<0.001	0.88	0.80–0.97	0.011
Educational level						
Primary school completed/Less than primary school/No formal schooling	Reference	-	-	Reference	-	-
Secondary school completed/High school (or equivalent) completed	1.15	1.06–1.25	0.001	1.08	1.00–1.17	0.065
University post-graduate degree completed/university completed/college/pre-university	0.98	0.90–1.07	0.695	1.20	1.10–1.31	<0.001
Living area						
City	Reference	-	-	Reference	-	-
Town	1.39	1.20–1.62	<0.001	1.29	1.12–1.48	<0.001
Village or rural area	1.5	1.36–1.66	<0.001	1.34	1.22–1.47	<0.001
Smoking						
No	Reference	-	-	Not included *		
Yes	0.97	0.91–1.05	0.481			
Compliance with physical distancing						
No	Reference	-	-	Reference	-	-
Yes	0.44	0.40–0.48	<0.001	0.46	0.42–0.50	<0.001
Compliance with mask use						
No	Reference	-	-	Reference	-	-
Yes	0.52	0.48–0.57	<0.001	0.66	0.61–0.70	<0.001
Food insecurity						
No	Reference	-	-	Not included *		
Yes	0.94	0.89–1.00	0.07			
Economic insecurity						
No	Reference	-	-	Reference	-	-
Yes	0.77	0.72–0.82	<0.001	0.80	0.75–0.85	<0.001
Anxiety symptomatology						
No	Reference	-	-	Reference	-	-
Yes	0.78	0.77–0.88	<0.001	0.87	0.82–0.92	<0.001
Depressive symptomatology						
No	Reference	-	-	Reference	-	-
Yes	0.82	0.77–0.88	<0.001	0.93	0.87–0.99	0.03
Comorbidities						
0	Reference	-	-	Reference	-	-
1	0.70	0.66–0.74	<0.001	0.82	0.77–0.87	<0.001
2 or more	0.66	0.61–0.72	<0.001	0.87	0.81–0.95	0.001
Vaccinated						
No	Reference	-	-	Reference	-	-
Yes	0.25	0.23–0.28	<0.001	0.26	0.23–0.28	<0.001
Had COVID-19						
No	Reference	-	-	Reference	-	-
Yes	0.91	0.84–0.98	0.014	0.93	0.87–0.99	0.045

95%CI: 95% confidence intervals; cPR: Crude prevalence ratio; aPR: Adjusted prevalence ratio.* Not included due to not having a statistically significant association in the crude model.

## Data Availability

Restrictions apply to the availability of these data. The authors obtained the data after signing a contract with the University of Maryland and have no permission to share the database.

## Supplementary Materials

The following are available online at https://www.mdpi.com/article/10.3390/vaccines9111303/s1, Table S1: Proportion of parents with intention to vaccinate their children against COVID-19 in Latin America and the Caribbean region, File S1: Questionnaire.
