# Supplementary material for: Prevalence and Factors Associated with Parents’ Non-Intention to Vaccinate Their Children and Adolescents against COVID-19 in Latin America and the Caribbean
Source: Vaccines (Basel). 2021 Nov 9;9(11):1303. doi: 10.3390/vaccines9111303 (PMC8624413; doi:10.3390/vaccines9111303)
Supplement: Supplementary file 1 [file vaccines-09-01303-s001.zip › vaccines-1368924-supplementary.pdf]

Table S1. Proportion of parents with intention to vaccinate their children against COVID-19 in Latin America and the Caribbean region.

	Parents' intention to vaccinate their children against COVID-19					
	Yes			No		
		Weighted proportion			Weighted proportion	
Countries	n	%	95%CI	n	%	95%CI
Argentina	19402	90.5	90.2-90.8	1850	9.5	9.2-9.8
Bolivia	4169	86.2	84.7-87.5	565	13.8	12.5-15.3
Brazil	79104	93.9	93.1-94.6	4409	6.1	5.4-6.9
Chile	8159	92.4	91.3-93.3	616	7.6	6.7-8.7
Colombia	15409	90.6	89.8-91.3	1476	9.4	8.7-10.2
Costa Rica	3295	92.6	90.5-94.3	220	7.4	5.7-9.5
Dominican Republic	2682	90.6	89.0-92.0	258	9.4	8.0-11.0
Ecuador	6027	90.6	88.1-92.7	514	9.4	7.3-11.9
El Salvador	2596	93.2	91.6-94.5	180	6.8	5.5-8.4
Guatemala	3486	89	87.5-90.4	403	11	9.6-12.5
Haiti	144	50	39.7-60.3	142	50	39.7-60.3
Honduras	1997	94.1	92.1-95.6	113	5.9	4.4-7.9
Mexico	43750	94.6	94.0-95.1	2200	5.4	4.9-6.0
Nicaragua	1486	87.2	85.4-88.7	217	12.8	11.3-14.6
Panama	1113	86.3	84.5-88.0	157	13.7	12.0-15.5
Paraguay	2305	91	88.5-93.1	197	10	6.9-11.5
Peru	7558	92.7	91.2-94.0	531	7.3	6.0-8.8
Puerto Rico, U.S.	1694	92	89.7-93.8	158	8	6.2-10.3
Uruguay	2941	87.1	85.7-88.4	397	12.9	11.6-14.3
Venezuela	5227	89.6	88.5-90.5	593	10.4	9.5-11.5

95%CI: 95% confidence intervals.

Supplementary file: Questionnaire

1. You understand the above and consent to take part in this survey run by the University of Maryland and Johns Hopkins University.
    a. Yes
    b. No
2. Do you consent with sharing your data with these academic institutions?
    a. Yes
    b. No
3. You must be 18 years or older to take this survey. Are you 18 years or older?
    a. Yes
    b. No
4. What is the country or region where you are currently staying?
    a. See country region response map file.
5. What is your gender?
    a. Male
    b. Female
    c. Other
6. What is your age?
    a. 18-24 years
    b. 25-34 years
    c. 35-44 years
    d. 45-54 years
    e. 55-64 years
    f. 65-74 years
    g. 75 years or older
7. What is the highest level of education that you have completed?
    a. No formal schooling
    b. Less than primary school
    c. Primary school completed
    d. Secondary school completed
    e. High school (or equivalent) completed
    f. College/ pre-university/ University completed
    g. University post-graduate degree completed
8. Which of these best describes the area where you are currently staying?
    a. City
    b. Town
    c. Village or rural area
9. As far as you know, have you ever had coronavirus (COVID-19)?
    a. Yes
    b. No
10. Have you had a COVID-19 vaccination?
    a. Yes
    b. No
    c. I don't know
11. In the past 7 days, how often did you intentionally avoid contact with other people?
    a. All of the time
    b. Most of the time
    c. Some of the time
    d. A little of the time

    e. None of the time

12. In the past 7 days, how often did you wear a mask when in public?
    a. All of the time
    b. Most of the time
    c. Some of the time
    d. A little of the time
    e. None of the time
    f. I have not been in public during the past 7 days
13. During the past 7 days, how often did you feel so nervous that nothing could calm you down?
    a. All of the time
    b. Most of the time
    c. Some of the time
    d. A little of the time
    e. None of the time
14. During the past 7 days, how often did you feel so depressed that nothing could cheer you up?
    a. All of the time
    b. Most of the time
    c. Some of the time
    d. A little of the time
    e. None of the time
15. How worried are you about having enough to eat in the next week?
    a. Very worried
    b. Somewhat worried
    c. Not too worried
    d. Not worried at all
16. How worried are you about your household's finances in the next month?
    a. Very worried
    b. Somewhat worried
    c. Not too worried
    d. Not worried at all
17. Have you ever been told by a doctor, nurse, or other health professional that you have any of the following medical conditions? Please select all that apply.
    a. Asthma
    b. Chronic lung disease such as COPD, chronic bronchitis, or emphysema
    c. Cancer
    d. Diabetes
    e. High blood pressure
    f. Kidney disease
    g. Weakened or compromised immune system
    h. Heart attack, heart disease, or other heart condition
    i. Obesity
    j. None of these
18. Do you smoke cigarettes?
    a. Yes
    b. No
19. Will you choose to get a COVID-19 vaccine for your child or children when they are eligible?
    a. Yes, definitely
    b. Yes, probably

    c. No, probably not
    d. No, definitely not
    e. I don't have children

20. In the past 24 hours, have you personally experienced any of the following symptoms?
    a. Chest pain
    b. Nausea
    c. Loss of smell or taste
    d. Headache
    e. Chills
    f. Fever
    g. Cough
    h. Difficulty Breathing
    i. Fatigue
    j. Stuffy or Runny nose
    k. Aches or muscle pain
    l. Sore throat
    m. Chest pain
    n. Nausea
    o. Loss of smell or taste
    p. Headache
    q. Chills
